# Acid sphingomyelinase/ceramide system in schizophrenia: implications for therapeutic intervention as a potential novel target

**DOI:** 10.1038/s41398-022-01999-7

**Published:** 2022-06-23

**Authors:** Chuanjun Zhuo, Feifei Zhao, Hongjun Tian, Jiayue Chen, Qianchen Li, Lei Yang, Jing Ping, Ranli Li, Lina Wang, Yong Xu, Ziyao Cai, Xueqin Song

**Affiliations:** 1 Key Laboratory of Real Time Tracing Brain Circuit, Tianjin Medical Affiliated Tianjin Fourth Center Hospital, Nankai University Affiliated Tianjin Fourth Center Hospital, Tianjin Fourth Hospital, 300140 Tianjin, China; 2 The key Laboratory of Psychiatric-Neuroimaging-Genetics and Comorbidity (PNGC_Lab) of Tianjin Anding Hospital, Tianjin Mental Health Center of Tianjin Medical University, 300222 Tianjin, China; 3 Brain Micro-imaging Center of Psychiatric Animal Model, Wenzhou Seventh Peoples Hospital, 325000 Wenzhou, China; 4 Department of Psychiatry, The Fourth Center Hospital of Tianjin Medical University, 300222 Tianjin, China; 5 Key Laboratory of the Macro-Brain Neuroimaging Center of Animal Model, Wenzhou Seventh Peoples Hospital, 325000 Wenzhou, China; 6 Department of Psychiatry, The First Hospital of Shanxi Medical University, 03000 Taiyuan, China; 7 Department of Psychiatry, First Affiliated Hospital of Zhengzhou University, 450052 Zhengzhou, China

**Keywords:** Pharmacology, Schizophrenia

## Abstract

Schizophrenia is a severe mental illness, as the efficacies of current antipsychotic medications are far from satisfactory. An improved understanding of the signaling molecules involved in schizophrenia may provide novel therapeutic targets. Acid sphingomyelinase (ASM) catalyzes cellular membrane sphingomyelin into ceramide, which is further metabolized into sphingosine-1-phophate (S1P). ASM, ceramide, and S1P at the cell surface exert critical roles in the regulation of biophysical processes that include proliferation, apoptosis, and inflammation, and are thereby considered important signaling molecules. Although research on the ASM/ceramide system is still in its infancy, structural and metabolic abnormalities have been demonstrated in schizophrenia. ASM/ceramide system dysfunction is linked to the two important models of schizophrenia, the dopamine (DA) hypothesis through affecting presynaptic DA signaling, and the vulnerability-stress-inflammation model that includes the contribution of stress on the basis of genetic predisposition. In this review, we highlight the current knowledge of ASM/ceramide system dysfunction in schizophrenia gained from human and animal studies, and formulate future directions from the biological landscape for the development of new treatments. Collectively, these discoveries suggest that aberrations in the ASM/ceramide system, especially in ASM activity and levels of ceramide and S1P, may alter cerebral microdomain structure and neuronal metabolism, leading to neurotransmitter (e.g., DA) dysfunction and neuroinflammation. As such, the ASM/ceramide system may offer therapeutic targets for novel medical interventions. Normalization of the aberrant ASM/ceramide system or ceramide reduction by using approved functional inhibitors of ASM, such as fluvoxamine and rosuvastatin, may improve clinical outcomes of patients with schizophrenia. These transformative findings of the ASM/ceramide system in schizophrenia, although intriguing and exciting, may pose scientific questions and challenges that will require further studies for their resolution.

## Introduction

Schizophrenia is a severe mental illness affecting approximately 20,000,000 people globally [1]. Despite their heterogeneity, symptoms are usually classified into three major categories: positive (hallucinations, delusions); negative (lack of motivation, apathy, or both); and cognitive [2]. Schizophrenia is associated with considerable disability and a 2- to 3-fold higher mortality rate than that of the general population, yet cure has been elusive [3]. Most antipsychotic medications act by blocking D2-type dopamine (DA) receptors, among other receptors, leading to partial responses in patients with schizophrenia. Despite their attenuation of the positive symptoms of schizophrenia, current antipsychotic drugs are generally poorly effective or even ineffective for the improvement of negative symptoms and cognitive impairments, and may also induce side effects. Indeed, the efficacy of currently available therapies is far from satisfactory, and mechanisms of action are more complex than dopamine D2 receptor blockade [4, 5]. As such, a better understanding of the pathophysiology is necessary to guide the development of more effective therapy.

The diverse structural and functional roles of membrane lipids have ignited interest in the roles of a wide range of lipid species, such as sphingolipids and phospholipids, in neuropsychiatric diseases that include schizophrenia, bipolar disorder, major depressive disorder, attention deficit hyperactivity disorder, and autism. Historically, the membrane hypothesis of schizophrenia led to exploratory studies of aberrant lipid species and advanced the understanding of the roles of several lipid molecules and related pathways. Recent advances in lipidomics and related technologies, such as mass spectrometry, that allow a more comprehensive and large-scale determination of lipids, molecular pathways, and networks; have greatly facilitated these studies.

Among the lipids associated with schizophrenia, sphingomyelin, a sphingolipid species, has gained increasing attention. Sphingomyelin is a critical component of the myelin sheath that vitally affects broad cerebral functions from cognition to movement. On the cell membrane, sphingomyelin is enzymatically catabolized by acid sphingomyelinase (ASM) to generate ceramide and phosphocholine [6]. Abnormal lipid species may affect cerebral connectivity, neuroinflammation, proliferation, and the formation of lipid rafts. Abnormalities of sphingomyelin and its metabolites, such as ceramide, have been associated with psychomorbidity as well as cognitive impairments in patients with schizophrenia [7].

In this review, we aimed to highlight findings of the ASM/ceramide system obtained through human and animal studies of schizophrenia; discuss ASM/ceramide system abnormalities in neuropathogenesis; and to propose future directions from the biological landscape for therapeutic development. In view of the potential roles of aberrant ASM/ceramide systems in the two important models of schizophrenia that include the dopamine (DA) hypothesis that implicates abnormal presynaptic DA signaling and the vulnerability-stress-inflammation model of the etiology of psychotic and cognitive symptoms experienced by patients with schizophrenia; the ASM/ceramide system could offer therapeutic targets for the development of new antipsychotic interventions. These transformative findings of the ASM/ceramide system in schizophrenia, although intriguing and exciting, may pose scientific questions and challenges in need of resolution through future studies.

## Acid sphingomyelinase/ceramide system

### ASM hydrolyses sphingomyelin to liberate ceramide and phosphorylcholine and create a cascade of bioactive lipids

ASM is one of six sphingomyelinases that include acidic, Mg(2+)-independent neutral, Mg(2+)-dependent neutral, alkaline, secretory, and bacterial sphingomyelinases [8]. ASM is comprised of the catalytic, N-terminal saposin, and proline-rich domains. Human sphingomyelinases are categorized into three types according to their optimal pH (acid, neutral and alkaline): ASM, neutral sphingomyelinase, and alkaline sphingomyelinase [9, 10]. ASM is the most studied of the three types of human sphingomyelinases, and is located primarily in the lysosome where optimal pH values range from 4.5 to 5.0 [6, 11, 12]. ASM can translocate to the cell surface during the constant recycling of lysosomes to the plasma membrane [6, 11, 12]. In both the lysosome and the cell membrane, ASM catalyzes sphingomyelin, an abundant sphingolipid on mammalian cellular membranes and the most abundant sphingolipid in eukaryotes, to release ceramide and phosphorylcholine [10, 13]. Notably, ASM exerts a critical role at the cell surface to control the biophysical properties of the cell membrane by catalyzing sphingomyelin to generate ceramide and other bioactive molecules [6, 10, 13].

The enzymatic activity of ASM is promoted by numerous stimuli, including pathogens (viruses and bacteria) and reactive oxygen species (ROS). In vitro studies have indicated that ASM is activated by the treatment of cells with hydrogen peroxide, but suppressed by ROS scavengers such as superoxide dismutase and N-acetylcysteine. Interestingly, oxidation of ASM at the cysteine residue 629 from the C-terminus stimulates its activation, which is in line with the effects of ROS and its scavengers [14, 15].

By contrast, genetic deficiency of ASM causes a Niemann–Pick disease (NPD) variant referred to as ASM-deficient NPD, in which sphingomyelin and cholesterol accumulate in cerebral lysosomes. NPD is fatal, with death occurring as early as 18 months of age. In addition to early death, clinical features of ASM-deficient NPD include progressive neurological degeneration and psychotic manifestations such as slurred speech; mental retardation; and loss of intellectual ability that usually leads to dementia. However, the aforementioned complications are absent in patients with other NPD phenotypes characterized by normal ASM activity.

### Sphingomyelin, ceramide, sphingosine, and sphingosine-1-phosphate function as structural and bioactive or signaling molecules

Like other sphingolipids, sphingomyelin has long been considered an essential structural molecule of cellular membranes. An increasing body of evidence illustrates the roles of sphingomyelin in physiological and pathological processes. In fact, ASM, sphingomyelin, and ceramide are critically involved in many aspects of cell signaling.

Ceramide is comprised of a sphingosine backbone covalently linked by an amide bond to a fatty acid, which confers high hydrophobicity. The paradigm-shifting work of Drs. Hannun, Obeid, and Kolesnick suggested for the first time that ceramide functions as an intracellular mediator [16–18]. Subsequently, a wealth of evidence has indicated that ceramide is an important lipid messenger. Ceramide molecules on the cell membrane aggregate spontaneously, leading to the formation of ceramide-enriched lipid microdomains (also termed ceramide-rich platforms) [11, 19, 20]. Notably, ceramide-enriched platforms play a crucial role in ASM/ceramide-mediated signaling pathways [11, 21].

Ceramide liberated from the hydrolysis of sphingomyelin regulates the clustering of membrane proteins via microdomain and lipid raft organization. A growing number of studies have demonstrated its roles in physiological and pathological processes. Ceramide-enriched microdomains on the cell surface reorganize, cluster, and aggregate important cell-surface receptors, such as CD95 (also known as Fas, Apo1), CD40 (a member in the tumor necrosis factor (TNF)-receptor superfamily), death receptor 5 (DR5, a pro-apoptotic cell-surface receptor) and β1-integrin [22–24]. In addition, ceramide-enriched microdomains mediate responses to various stress stimuli (e.g. ultraviolet light, γ -irradiation, viral and bacterial infections) [25–27]. Upon trapping and clustering in ceramide-enriched microdomains on the cell membrane, cell-surface receptors in proximity to signaling molecules consequently amplify signaling [28].

**Fig. 1:**
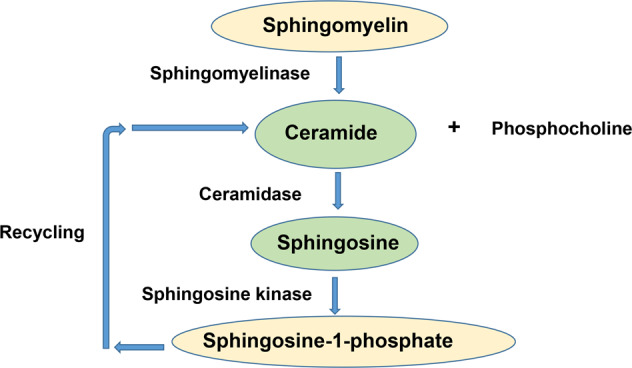
Catabolic pathway of sphingomyelin to generate ceramide and subsequent sphingosine-1-phophate. Acid sphingomyelinase (ASM) converts sphingomyelin to liberate ceramide and phosphocholine in the lysosome and on the plasma membrane. Ceramide is hydrolyzed by ceramidase to form sphingosine, which is subsequently phosphorylated by sphingosine kinase to generate sphingosine-1-phophate (S1P).

Ceramide in the cell membrane can be enzymatically hydrolyzed by ceramidase to generate sphingosine, which can be subsequently phosphorylated by sphingosine kinase to produce sphingosine-1-phophate (S1P) (Fig. 1). S1P is considered a critical regulator of inflammation [29]. In addition, some studies have indicated that S1P acts as a signaling molecule in the regulation of a broad range of cellular functions including the differentiation of oligodendrocytes; myelination; autophagy; and hematopoietic cell trafficking through interaction with an S1P receptor (S1PR) [30–32]. Five S1P receptors have been identified that include S1PR1, S1PR2, S1PR3, S1PR4, and S1PR5, of which four (S1PR1, S1PR2, S1PR3, and S1PR5) are expressed in the central nervous system [31, 32]. Interestingly, S1PR5 is expressed more abundantly in the frontal cortex and corpus callosum than the other three cerebral S1P receptors in mice and humans [31, 33]. A notable difference in S1PR1 and S1PR5 expressions was observed between humans and mice, with S1PR5 most abundantly expressed in the corpus callosum of humans and S1PR1 highly expressed in the frontal cortex and corpus callosum of mice [33]. Concordant with the results of O’Sullivan et al., a recent study demonstrated that the S1PR4 mRNA and protein expressions were undetectable in the frontal cortex and corpus callosum of both mice and humans [31, 33]. Although the brain contains the highest content of S1P in the human body, it is still unclear whether the S1P has the same importance for all cell types or if its functions are more specific to particular cell lines or tissues [34, 35]. With the existing discoveries of its roles in health and disease, further in-depth investigations are needed to gain insights into the mechanistic roles of S1P as well as the pathways that are mediated by its alterations, and to better understand the neurological causes of mental illnesses.

### Acid sphingomyelinase/ceramide system in schizophrenia

Abnormalities of sphingomyelin and ceramide in the ASM-ceramide signaling pathway are highly associated with schizophrenia, as evidenced by lipidomic studies of peripheral sub-tissues (circulating red blood cells [RBCs] and epidermis) and brain tissue [36–38].

High-throughput lipidomic profiling of peripheral blood samples have identified specific changes of lipids produced by the ASM-dependent catabolism of sphingomyelin (e.g., ceramide) in patients with schizophrenia [38]. Keshavan et al. [37] noted elevated sphingomyelin levels in RBC membranes of psychotic patients. Those with schizophrenia or schizoaffective disorder had significantly higher levels of RBC membrane sphingomyelin compared with healthy control subjects. Although this finding is not definitive evidence of causality, the abnormality observed in this early study [37] provided the foundation for later studies that have linked abnormal cerebral ASM/ceramide metabolism to the pathogenesis of schizophrenia. Ponizovsky et al. [36] correlated decreased erythrocytic levels of sphingomyelin with negative symptoms in patients with schizophrenia. Tessier et al. [7] performed a lipidomic analysis of RBC membrane phospholipid profiles to identify associated phospholipid molecular species and to determine whether abnormalities, including those of sphingomyelin and ceramide, may contribute to aberrant DA signaling in schizophrenia. Among membrane phospholipid abnormalities, sphingomyelin percentage was most robustly correlated with schizophrenia. When patients with schizophrenia were stratified into two subsets according to the RBC membrane sphingomyelin content i.e., low and high sphingomyelin membrane content groups (SCH c/SM− and SCH c/SM+ groups, respectively), the SCH c/SM− group had significantly higher positive, total, and cognitive/disorganized psychopathology scores on the Positive and Negative Syndrome Scale (a widely used instrument for rating symptoms of schizophrenia) and also demonstrated poor cognitive performance in comparison with those in the SCH c/SM+ group [7].

Consistent with the abnormal lipid profiles observed in RBCs, significantly increased ceramide levels have been demonstrated in skin samples from first-episode schizophrenia patients. Smesny et al. [39] showed significantly elevated ceramide levels and a higher ratio of ceramide to total lipids in the stratum corneum of first-episode schizophrenia patients compared to controls.

Lipidomic studies of cerebral tissue are more clinically relevant than those of peripheral sub-tissues such as circulating RBCs and epidermis, because neurolipidomic studies seek to understand dynamic alterations of membrane lipids in the brain and their relation to cerebral function. The role of abnormal cerebral ASM/ceramide metabolism in schizophrenia is supported by studies in rats [40]. The catabolism of sphingomyelin by ASM in rat primary hippocampal neurons can change phospholipid composition and therefore disrupt lipid rafts on the plasma membrane, leading to significant alterations in the desensitization kinetics of the alpha 7 nicotinic acetylcholine receptor (α7nAChR), presumably by affecting its anchoring in lipid rafts due to decreased sphingomyelin and increased ceramide [41]. α7nAchR is a member of the nAChR family and plays a critical neurotransmission by directly activating DA release through interactions with glutamatergic and GABAergic neurons. Furthermore, deficient α7nAchR function has been demonstrated in schizophrenia [41]. The regulatory role of sphingomyelin on α7nAchR activity was confirmed in rat primary hippocampal neurons by the same research group, by demonstrating that inhibition of sphingomyelin synthesis desensitized α7nAchR, while by co-treatment with sphingomyelin abrogated the effect [41]. Consequently, sphingomyelin supplementation or targeting the ASM/ceramide system may recover, at least to some extent, disrupted lipid rafts and α7nAchR function, and may thereby potentially benefit patients with schizophrenia.

The aforementioned in vivo results are consistent with reduced thalamic sphingomyelin levels in postmortem specimens from patients with schizophrenia [42]. Abnormal myelination has been observed by magnetic resonance imaging and postmortem analysis of brain tissues from patients with schizophrenia [7, 43, 44]. Postmortem studies of brain tissues from patients with schizophrenia have also indicated elevated ceramide levels, especially in white matter but not in gray matter [29, 33, 38].

Most recently, Esaki and colleagues examined sphingolipid levels of representative white matter (corpus callosum) and Brodmann area 8 of the frontal cortex in postmortem brain samples obtained from patients with schizophrenia, bipolar disorder, or major depressive disorder, and age-/sex-matched controls [33]. Levels of S1P were significantly lower in the corpus callosum in patients with schizophrenia, but not in those with major depressive or bipolar disorders in comparison with age- and sex-matched controls, suggesting that decreased S1P in cerebral white matter might be specific to schizophrenia [33]. Further studies have suggested that lower S1P levels in the corpus callosum of postmortem brain samples from patients with schizophrenia might be attributed to the schizophrenia-associated up-regulation of genes that encode S1P-degrading enzymes [33]. In addition, elevated ceramide and lowered S1P levels have been shown to promote apoptosis and induce cell-cycle arrest in postmortem brain samples of patients with schizophrenia [33]. These recent data suggest that abnormal S1P and ceramide levels may lead to structural and functional abnormalities of the brain white matter involved in schizophrenia.

Clinical and in vivo studies have revealed a link between antipsychotic medications and plasma membrane phospholipids [45–47]. Although antipsychotic drugs may alter plasma membrane lipid composition, human and animal studies found that the intake of haloperidol or risperidone was not significantly associated with lowered levels of S1P in the corpus callosum [33]. Esaki and colleagues also showed that there was no significant correlation between the doses of antipsychotic medications (haloperidol or risperidone) after conversion to chlorpromazine equivalents and S1P levels in schizophrenia patients receiving long-term antipsychotic therapy [33]. As such, in vivo and clinical data have excluded the possibility that antipsychotic medications lower S1P levels in schizophrenia, suggesting that abnormal S1P levels in schizophrenia are unlikely to be caused by antipsychotic mediations [33].

**Fig. 2:**
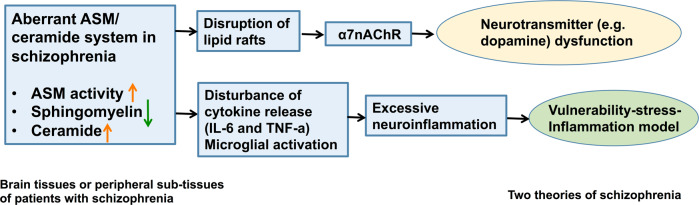
Hypothetical role of the acid sphingomyelinase/ceramide system in schizophrenia. Aberrant ASM activity and abnormal ceramide and sphingomyelin levels were observed in the brain and peripheral sub-tissues (red blood cells and epidermis) of patients with schizophrenia. ASM/ceramide system dysfunction may hypothetically perturb neurotransmission in the dopamine (DA) model through the desensitization kinetics of the alpha 7 nicotinic acetylcholine receptor (α7nAChR), and promote inflammation by upregulating interleukin-6 production and TNF-alpha signaling in the vulnerability-stress-inflammation model of schizophrenia.

Given that sphingomyelin and ceramide content at the membrane surface are controlled primarily by the ASM-ceramide system, ASM-ceramide signaling may influence presynaptic DA signaling and contribute to aberrant DA function in schizophrenia [7, 41, 43, 44] (Fig. 2).

In addition to the effects of sphingomyelin and ceramide on presynaptic DA release, the ASM-ceramide system may promote neuroinflammation through cytokine release, microglial activation, and other immune processes that have been demonstrated in schizophrenia [48–51] (Fig. 2). The vulnerability-stress-inflammation model, which includes the contribution of stress to schizophrenia on the basis of genetic susceptibility, is rapidly evolving in the field of schizophrenia research, and focuses on disturbances of cytokines and other inflammatory mediators potentially caused by pathogen exposures and infections [48, 51, 52]. ASM plays important roles in the regulation of interleukin-6 production and TNF-alpha signaling [53]. For example, the combination of prenatal maternal infection and subsequent and inflammation) and psychosocial stressors impact DA and glutamate transmission [54]. Immune activation has been directly implicated in schizophrenia as evidenced by the presence of central nervous system inflammation after local and systemic infection; inflammatory cytokines released by microglia (the innate immune cells of the brain) in response to infection; and overactivation of microglia [52, 55–57]. Ceramide-rich platforms mediate responses to a variety of stressors such as intracellular bacterial and viral infections [12, 58].

Increased oxidative stress (OS) in acute schizophrenia and disturbed ceramide metabolism may be byproducts of lipid membrane peroxidation. This hypothesis is concordant with findings of a genetic abnormality of antioxidant defense in patients with schizophrenia. In addition, Schwarz and colleagues associated elevated OS with glutamate/DA deficiency in patients with schizophrenia. These studies suggest that the Cer-SM pathway could be involved in the regulation of inflammatory response and immune function [38].

Future in vivo investigations in pharmacological, genetic, lesion, and developmental rodent models, and clinical studies are needed to gain further insight into the underlying mechanisms of schizophrenia. The aforementioned studies of RBCs, epidermis, and brain tissues have identified abnormalities of specific lipid species of sphingomyelin and ceramide and their associations with symptoms and cognitive performance of patients with schizophrenia, and suggest that ASM, ceramide, sphingomyelin, and S1P may represent therapeutic targets.

### Future directions: The ASM/ceramide system as therapeutic target

The associations of the ASM/ceramide system, its signaling pathways, and schizophrenia have important implications for drug development. Unique therapeutic modalities may include the normalization of ASM/ceramide system function and ceramide levels. A class of approved medications that act as functional inhibitors of ASM (FIASMA) includes hydroxyzine and fluvoxamine, an antidepressant widely used to treat obsessive compulsive disorder and depression. FIASMAs induce the detachment of ASM from inner lysosomal membranes and thereby cause its consecutive inactivation [59]. However, clinical trials of adjunctive therapy using the FIASMA-antidepressant fluvoxamine to treat schizophrenia have given inconsistent results [60–62]. Because the activated ASM/ceramide system may be inhibited by approved FIASMAs and particularly by the antidepressants in this drug class, FIASMAs may be expected to benefit patients with schizophrenia or combined depressive disorders who have ASM/ceramide system dysfunction and abnormal ceramide levels. However, clinical evaluations of FIASMAs for the treatment of schizophrenia are lacking. Because of the exciting discoveries of aberrant ASM/ceramide system activation in the pathogenesis of common mood symptoms of depression (e.g., sadness, feeling worthless and hopeless) or combined depression in patients with schizophrenia, as well as low cost, it may be worthwhile to explore whether adding a low dose of a FIASMA antidepressant such as fluvoxamine to an antipsychotic agent could enhance clinical outcomes. A controlled clinical trial could compare FIASMA-antidepressant and non-FIASMA-antidepressant groups.

In addition to ASM inhibition with FIASMAs, the repurposing of rosuvastatin to reduce ceramide levels is expected to benefit patients with schizophrenia. Rosuvastatin belongs to the statin class of anti-hyperlipidemic–hydroxymethylglutaryl-coenzyme A inhibitors [63]. Furthermore, rosuvastatin reduces blood levels of ceramide. Adjunctive rosuvastatin improved the efficacy of olanzapine on behavioral impairment and hippocampal metabolic abnormalities in isolation-reared male rats [63]. Rosuvastatin also improved antipsychotic-induced dyslipidemia in patients with schizophrenia [63, 64]. In consideration of the high prevalence of metabolic syndrome that includes overweight, obesity, and hyperlipidemia in schizophrenia patients, rosuvastatin might bring multiple benefits. Consequently, studies to determine whether rosuvastatin therapy can improve the clinical outcomes of patients with schizophrenia may be worthwhile. Whether lowering ceramide levels with rosuvastatin may benefit mental health and manage hyperlipidemia could be investigated by comparing two groups of patients taking the antipsychotics with or without the addition of rosuvastatin.

## Conclusions

We have summarized structural and metabolic abnormalities of the ASM/ceramide system and their potential involvement in schizophrenia. The aberrant ASM/ceramide system; featuring ASM hyperactivity as well as abnormal levels of sphingomyelin, ceramide, and S1P; has been linked to two theories of schizophrenia, the DA and vulnerability-stress-inflammation models; and in turn to psychotic symptoms and cognitive impairments. In the context of the vulnerability-stress-inflammation model, viral infection and cytokine production are jointly regulated by the ASM/ceramide system. The normalization of ASM/ceramide system dysfunction and related expressions of bioactive molecules (e.g., ceramide, S1P) on the cell surface through pharmacological intervention is expected to benefit patients with schizophrenia. FIASMA-antidepressants are approved, well-tolerated, safe, and inexpensive drugs for the treatment of depression, obsessive compulsive disorder, or both. They act, at least in part, through down-regulation of the ASM/ceramide system. We suggest that the FIASMA-antidepressants may thus be expected to benefit patients with schizophrenia. Considering the inconsistent results among previous clinical trials and investigations on the FIASMA antidepressant fluvoxamine adjunctive therapy for patients with schizophrenia [60–62], it is worthy investigating whether FIASMAs may have therapeutic effect on a group of patients with schizophrenia or combined depressive disorders who present abnormal ASM/ceramide system. We also propose future directions from the biological landscape for the development of new treatments for schizophrenia. Collectively, these discoveries suggest that aberrations in the ASM/ceramide system, especially in ASM activity and levels of ceramide and S1P, may alter microdomain structure and dysregulate cerebral metabolic pathways, leading in turn to neurotransmitter (e.g., DA) dysfunction and neuroinflammation. Taken together, studies of the ASM/ceramide system may further the understanding of the signaling molecules involved in schizophrenia and guide the development of better therapeutic interventions. These transformative findings of the ASM/ceramide system in schizophrenia, although intriguing and exciting, may pose scientific questions and challenges that will require future studies for their resolution.
